# *Molossusmelini* Montani et al. 2021 (Chiroptera, Molossidae) in Brazil: new insights for distribuition, morphology and genetics

**DOI:** 10.3897/BDJ.12.e114261

**Published:** 2024-02-12

**Authors:** Ana Priscila Medeiros Olímpio, Amanda Cristiny da Silva Lima, Samira Brito Mendes, Beatriz Dybas da Natividade, Elmary da Costa Fraga, Maria Claudene Barros, Iracilda Sampaio

**Affiliations:** 1 Federal University of Pará, Belém, Brazil Federal University of Pará Belém Brazil; 2 Maranhão State University, São Luís, Brazil Maranhão State University São Luís Brazil; 3 Federal University of Paraíba, João Pessoa, Brazil Federal University of Paraíba João Pessoa Brazil; 4 Maranhão State University, Caxias, Brazil Maranhão State University Caxias Brazil

**Keywords:** Curitiba, Paraná, Atlantic Forest ecoregion, new register, Melin’s mastiff bat

## Abstract

**Background:**

The species *M.melini* has been observed in both the Pampa and Spinal ecoregions of Argentina. Researchers have underscored that distinguishing *M.melini* from other species within the same genus relies primarily on craniometric and molecular analyses. Morphological measurements alone do not offer a clear differentiation between *M.melini* and other members of this genus.

This study aims to document the presence of *M.melini* within the Brazilian ecoregion, focusing on its morphological, morphometric and genetic characteristics. By undertaking a comprehensive examination, we seek to contribute valuable insights into the distribution and differentiation of *M.melini* in this region.

**New information:**

*Molossusmelini* specimens exhibited a forearm length ranging from 39.9 to 40.08 mm. The average intraspecific divergence was 1.2%, with specimens from the Argentine Pampas clustering in the same clade with a 98% bootstrap support and a posterior probability of: Regarding dorsal colouration, the specimens displayed fur with two bands—a Snow White base colour and apex colours ranging from Olive Brown, Broccoli Brown, Wood Brown to Yellowish-Brown. This marks the first record of *M.melini* in Brazil, expanding its distribution 1,300 km northeastwards into the Curitiba, Paraná, Atlantic Forest Ecoregion. The findings contribute valuable information on the distribution, morphology, morphometrics and genetics of this species.

## Introduction

The genus *Molossus* stands out as one of the most diverse within the family Molossidae (Loureiro et al. 2018). Its species are widely distributed across the Neotropical Region, spanning from northern Mexico to southern Argentina and encompassing numerous Caribbean islands (López-González and Presley 2001, Loureiro et al. 2018). However, data on the distribution of several species within this genus remain limited, with notable gaps in sampling, particularly within the Brazilian territory, as highlighted by Loureiro et al. (2018).

As of the latest research, 15 *Molossus* species have been identified through molecular, morphological and morphometric analyses (Lim 2017, Loureiro et al. 2018, Loureiro et al. 2019, Montani et al. 2021). These species include:*M.molossus* (Pallas, 1766); *M.rufus* É. Geoffroy St.-Hilaire, 1805; *M.aztecus* De Saussure, 1860; *M.fluminensis* Lataste, 1891; *M.nigricans* Miller, 1902; *M.pretiosus* Miller, 1902; *M.currentium* Thomas, 1901; *M.bondae* Allen, 1904; *M.coibensis* Allen, 1904; *M.sinaloae* Allen, 1906; *M.verrilli* Allen, 1908; *M.milleri* Johnson, 1952; *M.alvarezi* González-Ruiz et al., 2011; *M.fentoni* Loureiro et al., 2018 and *M.melini* Montani et al., 2021. Importantly, the seven species have been documented within Brazilian ecoregions (Garbino et al. 2022).

*Molossusmelini*, specifically, was first described by Montani et al. (2021) in the Province of Santa Fe, Argentina, within the Pampa ecoregion. Pavé et al. (2023) subsequently expanded its known range by 230 km to the northeast, into the Spinal ecoregion of Argentina. Notably, there are no records of this species within Brazil to date. Researchers have emphasised the reliability of distinguishing *M.melini* from other genus members through craniometric and molecular analyses, as external measurements alone do not provide a clear distinction.

The molecular approach known as DNA barcoding, utilising the mitochondrial gene Cytochrome c Oxidase subunit I (COI), has gained widespread use for species identification (Hebert et al. 2003). It has prompted a re-evaluation of bat species in tropical regions, as observed in studies by Clare et al. (2011). One advantage of DNA barcoding is its association with an online database, BOLD: The Barcode of Life Data Systems (Gager et al. 2016), enabling sequence comparisons for similarity indices to aid in species identification. In this context, our research aims to document the occurrence of *M.melini* in Brazil, Paraná State, Curitiba City, Atlantic Forest Ecoregion. This will be accomplished through a comprehensive analysis encompassing morphological, morphometric and genetic aspects, employing DNA barcoding.

## Materials and methods

### Morphological Analyses

This study involved a detailed examination of the skin and skulls of four *Molossus* sp. specimens obtained from Curitiba, Paraná, Brazil (-25.4277800 and -49.2730600). These specimens, vouchers DZUP/CCMZ 2336, DZUP/CCMZ 2337, DZUP/CCMZ 2338 and DZUP/CCMZ 2339, are part of the mammal collection at UFPR. They were collected from various residences throughout the city. Our analysis encompasses a comprehensive set of external and cranial measurements and the results are compared with the findings of Montani et al. (2021) and Pavé et al. (2023) concerning *M.melini*.

A total of five external measurements were taken, encompassing the total length of the body (ToL), tail length (TL), hindfoot length (HFL), ear length (EL) and forearm length (FA). Additionally, 13 cranial measurements were recorded, including the greatest length of the skull including incisors (GLS), condylobasal length (CBL), breadth of the braincase (BB), mastoideal breadth (MB), zygomatic breadth (ZB), palatal length (PL), width across molars (M2–M2), width across canines (C–C), height of the sagittal crest (SAR), postorbital constriction (PC), length of the maxillary toothrow (LMxT), length of the mandible (LM) and length of the mandibular toothrow (LmdT). Of the specimens analysed, three were females and one was male. It is noteworthy that the skull of one female (DZUP/CCMZ 2336) was damaged and, consequently, excluded from the analyses. The qualitative characterisation of cranial characters followed the approach outlined by Loureiro et al. (2018). Furthermore, specimen colouration (code) was meticulously analysed and determined in accordance with Syme (1821).

### Molecular identification: obtaining of genetic marker and DNA sequencing

The total of DNA was extracted from muscle tissue using the Wizard Genomic DNA Purification Kit from Promega, following the manufacturer's recommended protocols. Specifically, we targetted the mitochondrial gene Cytochrome c Oxidase subunit I (COI) for isolation and amplification through a Polymerase Chain Reaction (PCR), as described by Folmer et al. (1994). The PCR cycling conditions were as follows: an initial denaturation step at 94°C for 3 minutes, followed by 40 cycles consisting of denaturation at 94°C for 45 seconds, annealing at 48°C for 45 seconds, extension at 72°C for 1 minute and 30 seconds and a final extension at 72°C for 3 minutes. Subsequently, the PCR products were purified and subjected to DNA sequencing using the Sanger sequencing method, as outlined by Sanger et al. (1977), with the use of an ABI™ 3500 automatic capillary sequencer. The sequences obtained will be deposited in GenBank, contributing to the body of genetic data available for future research in this field.

### Database and genetic analyses

**Figure 1.**
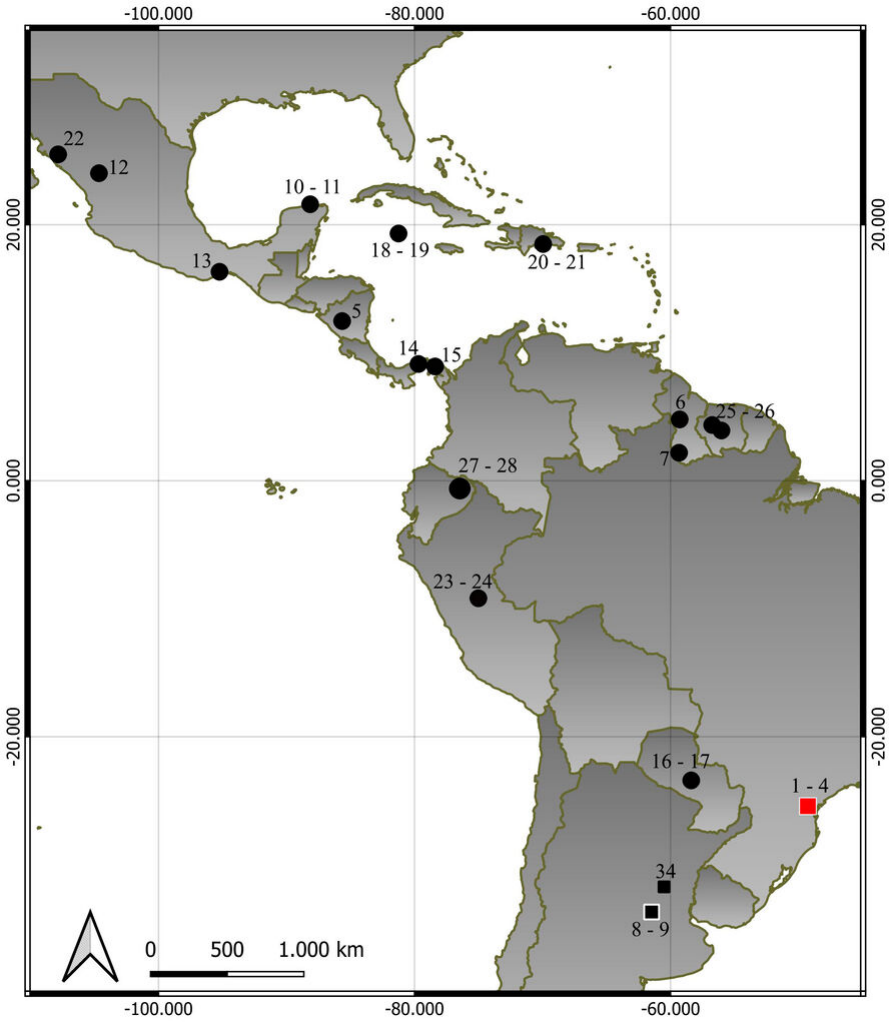
The map illustrates the localities of the *Molossus* specimens included in the current study. Additionally, black squares pinpoint the known occurrence locations of *M.melini*, while the red square signifies the inaugural record of *M.melini* in Curitiba, PR, Brazil, within the Atlantic Forest. The black square with white borders indicates the type locality of *M.melini* in Santa Fe Province, Argentina. The numbers correspond to the localities in Table 1 and number 34 corresponds to the record from Pavé et al. (2023), which lacks a COI sequence and, therefore, is not included in the table.

The database utilised for our analyses comprised 33 sequences (Table 1), encompassing a total of 657 base pairs. These sequences were sourced from both GenBank and the BOLD Systems platforms and represented a variety of species, 13 out of 15 *Molossus* species: *M.molossus*, *M.coibensis*, *M.aztecus*, *M.rufus*, *M.pretiosus*, *M.currentium*, *M.bondae*, *M.milleri*, *M.fentoni*, *M.verrilli*, *M.sinaloae*, *M.alvarezi* and *M.melini*. Our analysis incorporated samples collected from multiple geographic locations, including Ecuador, Peru, Mexico, Suriname, Nicaragua, Paraguay, Panama, Argentina, Brazil, the Cayman Islands, Guyana and the Dominican Republic. Additionally, outgroup sequences from the species *Eumopsauripendulus*, *Promopscentralis*, *Cynomopsabrasus*, *Nyctinomopslaticaudatus* and *Molossopstemminckii*, obtained from GenBank, were included in our study (see Fig. 1 and Table 1 for details). The specimens on the map (Fig. 1) are marked with numbers corresponding to those in Table 1. Specifically, number 34 corresponds to the entry from Pavé et al. (2023); however, it is not included in Table 1 due to the absence of a COI sequence. To delve deeper into the sequences generated for the *M.melini* specimens, we performed a similarity index analysis by plotting them on the BOLD Systems platform (https://www.boldsystems.org/index.php).

The sequences underwent meticulous inspection and editing using BioEdit software (Hall 1999). Regions of low quality were excluded as necessary to ensure data integrity. Alignment was carried out employing the default parameters of the Clustal W computer programme (Thompson et al. 1994). For the subsequent analysis, the average nucleotide divergence and phylogenetic relationships amongst samples were determined using Maximum Likelihood (ML). The evolutionary model GTR, incorporating the gamma rate parameter (G) and proportion of invariant sites (I), was applied in MEGA X (Kumar et al. 2018). The statistical robustness of the Maximum Likelihood tree was assessed through bootstrap analysis, involving 1000 replications (Felsenstein 1985).

Bayesian Inference (BI) was generated using the MrBayes 3.2.2 computer programme (Ronquist et al. 2012). Bayesian analysis was performed with Markov Chain Monte Carlo (MCMC) simulations run independently twice for 500,000 generations with four simultaneous chains, using a sampling frequency of 1,000. Convergence and effective sample sizes (ESS > 200) were checked using the programme Tracer v.1.7 (Rambaut et al. 2018) and the first 20% of trees were discarded as burn-in. A consensus tree was generated from the remaining trees. The inferred tree was visualised using FigTree v.1.4.4 software (http://tree.bio.ed.ac.uk/software/figtree/) and nodes were considered significantly supported when the posterior probabilities (PP) were above 0.80.

## Taxon treatments

### Molossus melini

Montani et al., 2021

92BE69D5-6131-518B-B74A-B8B199E6B485

#### Materials

- **Type status:** Other material. **Occurrence:** individualCount: 1; sex: female; lifeStage: adult; preparations: skin | skull | tissue (70% alcohol); photograph; DNA; associatedSequences: OR143786; occurrenceID: 860E0771-0BD9-57E5-A5C3-E0E8EC00533F; **Taxon:** scientificName: *Molossusmelini* Montani et al., 2021; kingdom: Animalia; phylum: Chordata; class: Mammalia; order: Chiroptera; family: Molossidae; genus: Molossus; specificEpithet: *melini*; taxonomicStatus: accepted; **Location:** continent: America; country: Brazil; stateProvince: Paraná; municipality: Curitiba; **Event:** year: 2020; month: 11; day: 05; **Record Level:** institutionID: Mammal Collection of Federal University of Paraná; institutionCode: DZUP/CCMZ 2336; basisOfRecord: PreservedSpecimen
- **Type status:** Other material. **Occurrence:** individualCount: 1; sex: female; lifeStage: adult; preparations: skin | skull | tissue (70% alcohol); photograph; DNA; associatedSequences: OR240233; occurrenceID: CAF91EE7-1352-5856-AAB7-1DE8E1FE01D4; **Taxon:** scientificName: *Molossusmelini* Montani et al., 2021; kingdom: Animalia; phylum: Chordata; class: Mammalia; order: Chiroptera; family: Molossidae; genus: Molossus; specificEpithet: *melini*; taxonomicStatus: accepted; **Location:** continent: America; country: Brazil; stateProvince: Paraná; municipality: Curitiba; **Event:** year: 2020; month: 11; day: 21; **Record Level:** institutionID: Mammal Collection of Federal University of Paraná; institutionCode: DZUP/CCMZ 2337; basisOfRecord: PreservedSpecimen
- **Type status:** Other material. **Occurrence:** individualCount: 1; sex: female; lifeStage: adult; preparations: skin | skull | tissue (70% alcohol); photograph; DNA; associatedSequences: OR144072; occurrenceID: 06575217-4228-5CBD-8094-CB152F9D4B90; **Taxon:** scientificName: *Molossusmelini* Montani et al., 2021; kingdom: Animalia; phylum: Chordata; class: Mammalia; order: Chiroptera; family: Molossidae; genus: Molossus; specificEpithet: *melini*; taxonomicStatus: accepted; **Location:** continent: America; country: Brazil; stateProvince: Paraná; municipality: Curitiba; **Event:** year: 2020; month: 11; day: 10; **Record Level:** institutionID: Mammal Collection of Federal University of Paraná; institutionCode: DZUP/CCMZ 2338; basisOfRecord: PreservedSpecimen
- **Type status:** Other material. **Occurrence:** individualCount: 1; sex: male; lifeStage: adult; preparations: skin | skull | tissue (70% alcohol); photograph; DNA; associatedSequences: OR144071; occurrenceID: 9E536082-BEBA-5AD0-947E-4238EFA59E70; **Taxon:** scientificName: *Molossusmelini* Montani et al., 2021; kingdom: Animalia; phylum: Chordata; class: Mammalia; order: Chiroptera; family: Molossidae; genus: Molossus; specificEpithet: *melini*; taxonomicStatus: accepted; **Location:** continent: America; country: Brazil; stateProvince: Paraná; municipality: Curitiba; **Event:** year: 2020; month: 02; day: 03; **Record Level:** institutionID: Mammal Collection of Federal University of Paraná; institutionCode: DZUP/CCMZ 2339; basisOfRecord: PreservedSpecimen

#### Diagnosis

**Figure 2.**
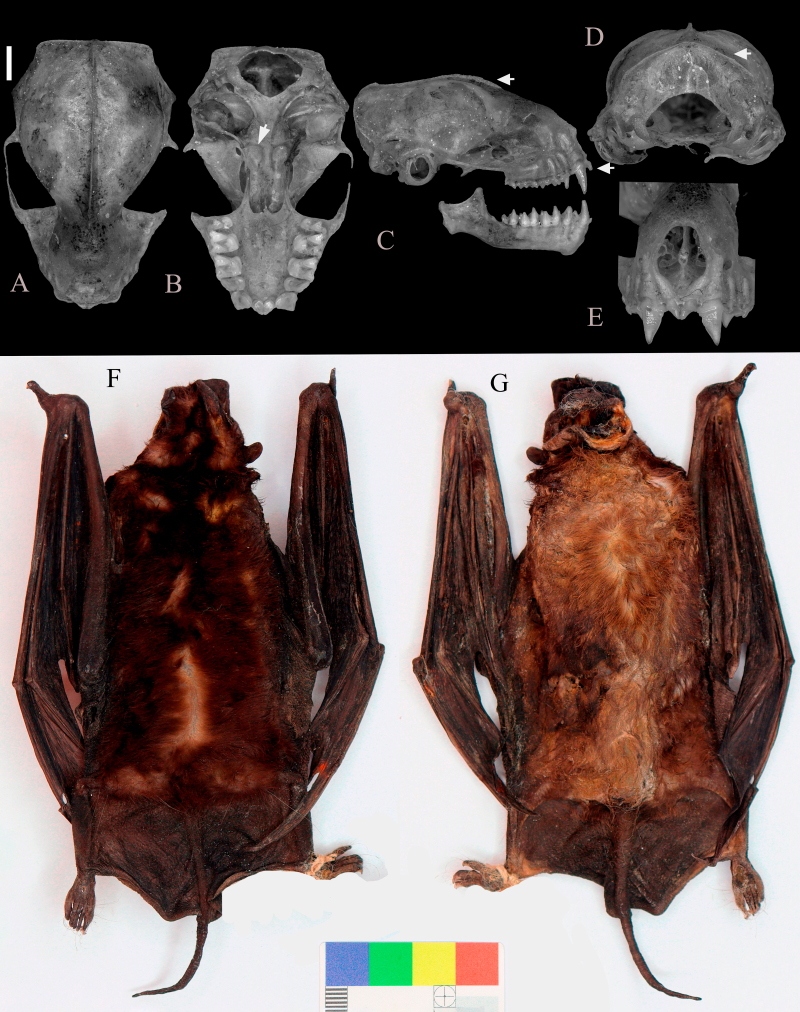
Female specimen of *Molossusmelini* (DZUP/CCMZ 2338) from Brazil, Curitiba, PR, Atlantic Forest. Views of the skull: **A** Superior; **B** Inferior, the arrow indicates the basiesphenoid pit; **C** Lateral, the arrows show the poorly-developed sagittal crest and well-projected incisors; **D** Posterior, the arrow shows the rounded shape of the occipital complex; **E** Frontal, highlighting the convergent incisors with the pincer-like tips; **F, G** Specimen's skin dorsal and ventral view respectively. Scale of 2 mm.

The *M.melini* specimens displayed a dorsal and ventral fur measuring 4 mm in length, each adorned with two colour bands. The colouration of the dorsal fur's base occupied 1/4 of its length, while, for the ventral fur, the base colour covered 1/5 of the fur. The variations in colouration amongst the *M.melini* specimens are detailed as follows: DZUP/CCMZ 2336 - Dorsal: base is Snow White (code 1) and the tip is Olive Brown (code 109). Ventral: base Snow White (code 1) and the tip is Broccoli Brown (code 108). DZUP/CCMZ 2337 - Dorsal: base is Snow White (code 1) and the tip is Wood Brown (code 105). Ventral: base Snow White (code 1) and the tip is Broccoli Brown (code 108). DZUP/CCMZ 2338 - Dorsal: base is Snow White (code 1) and the tip is Yellowish-Brown (code 104). Ventral: base Snow White (code 1) and the tip is Broccoli Brown (code 108) (Fig. 2F and G). DUZP/CCMZ 2339 - Dorsal: base is Snow White (code 1) and the tip is Olive Brown (code 109). Ventral: base Snow White (code 1) and the tip is Broccoli Brown (code 108).

The specimens exhibited a braincase larger than the rostrum and, in lateral view, the upper incisors converged with pincer-like tips projecting beyond the canines. The infraorbital foramen opened laterally in the rostral view. The basiesphenoid pit was rounded, well-defined and moderately deep, while the mastoid process was slightly directed towards the foramen. The sagittal and lambdoidal crests were poorly developed and the occipital complex had a rounded shape. The nasal process was well-developed and projected over the nasal cavity (refer to Fig. 2). For detailed external and cranial measurements of the *M.melini* specimens, including comparisons with data from Montani et al. (2021) and Pavé et al. (2023), please refer to Table 2. Ventrally, hairs extend across the wing membranes.

#### Distribution

Estancia Laguna San Carlos, approximately 10 km south of Melincué, General López Department, Santa Fe Province, Argentina (33°45'3.5"S; 61°24'25.2"W; 90 m elev.) (Montani et al. 2021) . Entre Rios Province, Parana Department, Parana City, Argentina (-31.747, -60.522; 77 m elev.) (Pavé et al. 2023) .

#### Biology

**Figure 3.**
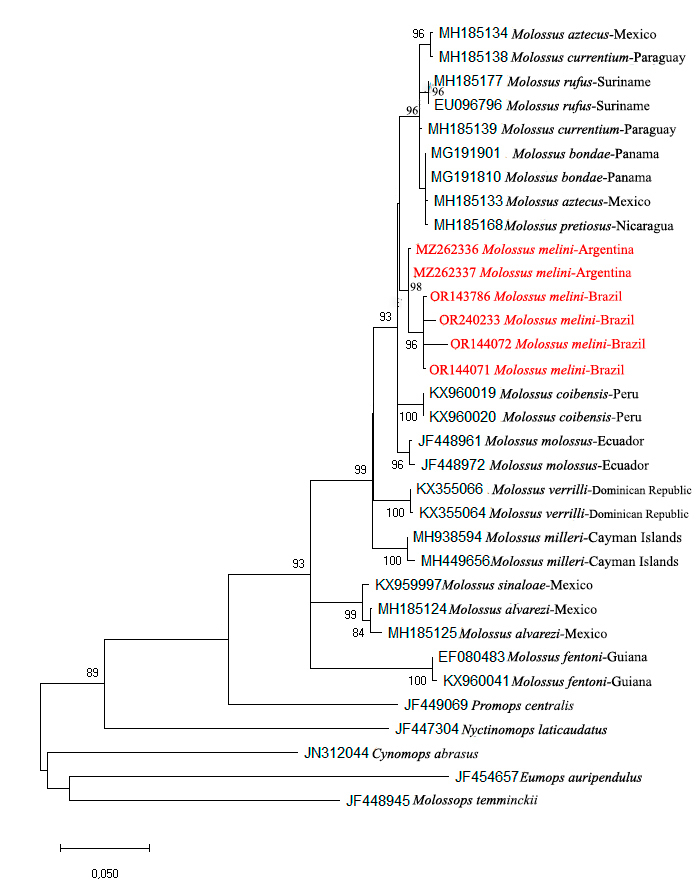
Maximum Likelihood for species of the genus *Molossus*, based on 657 base pairs of the mitochondrial gene COI: The analysis focuses on highlighting specimens of *Molossusmelini* from Brazil and Argentina. The values displayed on the branches represent the bootstrap values, with only those exceeding 80% being shown.

**Genetic**: Molecular analysis revealed a strong clustering of specimens from the present study with *M.melini* from the Argentine Pampas (GenBank), supported by a 98% bootstrap (refer to Fig. 3) and a posterior probability of 1 (Suppl. material 1). The average genetic divergence was found to be 1.2%. Notably, when plotting the sequences of the specimens on the BOLD Systems platform, a high level of similarity, ranging from 99.22% to 99.53%, was observed with deposited *M.melini* specimens. Consequently, this study marks the first record of *M.melini* for Brazil, extending its distribution more than 1,300 km northeastwards.

#### Taxon discussion

*Molossusmelini* was initially described by Montani et al. (2021) in the Pampa Region of Argentina, with the type locality identified as Estancia Laguna San Carlos, approximately 10 km south of Melincué, General López Department, Santa Fe Province, Argentina. Pavé et al. (2023) subsequently expanded the distribution of this species by 230 km northeast, documenting its presence in the Espinal Ecoregion of Argentina.

Currently, there is limited knowledge regarding the biology and ecology of *M.melini*. In this study, we contribute to the understanding of this species by documenting its occurrence, marking the first record in Brazilian territory. This discovery extends its distribution more than 1,300 km northeastwards. Our research delves into external and cranial morphology, as well as genetic aspects of *M.melini*. Prior to our study, seven *Molossus* species were documented in Brazil: *M.rufus*, *M.molossus*, *M.coibensis*, *M.aztecus*, *M.currentium*, *M.fluminensis* and *M.pretiosus* (Garbino et al. 2022).

Montani et al. (2021) highlighted morphological characters, such as orange colouration and specific cranial and body measurements, for the identification of *M.melini*. Subsequently, Pavé et al. (2023) emphasised the significance of orange colouration, forearm length (> 41 mm) and a combination of measurements as useful identification criteria. In our study, *M.melini* specimens exhibited forearm lengths ranging from 39.9 to 40.08 mm, slightly smaller than reported in the literature. Dorsal colouration presented two bands, a Snow White base colour and apex colours ranging from Olive Brown, Broccoli Brown, Wood Brown to Yellowish-Brown. This variation may be associated with a latitudinal gradient, a phenomenon observed in other mammals (Bogdanowicz 1990, Storz et al. 2001, Watt et al. 2010, Clauss et al. 2013).

Montani et al. (2021) suggested that *M.melini* shares overlapping measurements only with *M.alvarezi* and *M.currentium*. However, our research reveals morphological variation in forearm size that overlaps with that of other species, *M.aztecus*, *M.bondae*, *M.milleri*, *M.molossus* and *M.verrilli*, posing challenges for species identification. In the specimens of *M.melini* analysed, we observed that, ventrally, hairs extend across the wing membranes, a characteristic that is less evident in other species within the genus.

Furthermore, we observed that some diagnostic cranial characteristics of the species diverged when compared to the original description. Our specimens exhibited a less developed sagittal crest and lambdoidal crest and a rounded occipital complex, in contrast to the findings of Montani et al. (2021), who observed a triangular occipital complex in posterior view and well-developed sagittal and lambdoidal crests. It is important to note that few individuals have been analysed in the aforementioned studies, highlighting the need to include more specimens to understand the diagnostic characters of the species. It is worth noting that, morphologically, many species within *Molossus* are highly similar, complicating the identification and delimitation of species, as noted by Loureiro et al. (2018).

Genetic data indicate low intraspecific variation (1.2%) and a robust clustering of *M.melini* specimens from Brazil with those from the Pampa Region of Argentina. Additionally, when plotted in the BOLD Systems database, they exhibited high similarity ranging from 99.22% to 99.53% with *M.melini* specimens. This study contributes to documenting the occurrence of *M.melini* in Brazil, marking the third known locality and the first in Brazilian territory. It provides valuable insights into the distribution, morphology, morphometrics and genetics of this species.

## Supplementary Material

XML Treatment for Molossus melini

4C2F8C78-40C9-558C-8F79-3C020E9DFB1C 10.3897/BDJ.12.e114261.suppl1 Supplementary material 1 Bayesian Inference Data type phylogenetic Brief description Bayesian Inference for species of the genus *Molossus* based on 657 base pairs of the mitochondrial gene COI: The analysis focuses on highlighting specimens of *Molossusmelini* from Brazil and Argentina. The values on the branches represent the posterior probability (PP) and only values with PP > 0.80 are shown. File: oo_942870.jpg https://binary.pensoft.net/file/942870 Olímpio, A. P. M.

## Figures and Tables

**Table 1.** Locality, species and access codes from the BOLD Systems and GenBank online platforms and the field code of the samples in the present study for the *Molossus* specimens included in the analyses are as follows. Specimens marked with an asterisk (*) were initially identified in GenBank as *Molossusmolossus* and *Molossus* sp.; however, as per the identification by Loureiro et al. (2019), they were reclassified as *Molossusm.verrilli* (Dominican Republic) and *Molossusfentoni* (Guyana). The numbers in the map column correspond to the localities in Fig. 1.

**Map**	**Species**	**Specimen**	**Locality**	**BOLD Systems**	**GenBank**
1	*Molossusmelini*	DZUP/CCMZ 2336	Brazil, Paraná, Curitiba		OR143786
2	*Molossusmelini*	DZUP/CCMZ 2337	Brazil, Paraná, Curitiba		OR240233
3	*Molossusmelini*	DZUP/CCMZ 2338	Brazil, Paraná, Curitiba		OR144072
4	*Molossusmelini*	DZUP/CCMZ 2339	Brazil, Paraná, Curitiba		OR144071
5	*Molossuspretiosus*	TTU29780	Nicaragua, Boaco	GBGC16005-19	MH185168
6	Molossusfentoni*	ROM 109176	Guyana, Potaro-Siparuni	BCBNT388-06	EF080483
7	Molossusfentoni*	ROM:Mamm 122583	Guyana, Bototo Wau		KX960041
8	*Molossusmelini*	MG-ZV-M 338	Argentina, Santa Fe, Melincué		MZ262336
9	*Molossusmelini*	MG-ZV-M 339	Argentina, Santa Fe, Melincué		MZ262337
10	*Molossusalvarezi*	ROM F49115	Mexico	GBGC15864-19	MH185124
11	*Molossusalvarezi*	ROM F49121	Mexico, Yucatán	GBGC15868-19	MH185125
12	*Molossusaztecus*	CRD2494	Mexico, Durango	GBGC15112-19	MH185133
13	*Molossusaztecus*	TTU 33531	Mexico, Tehuantepec	GBGC16007-19	MH185134
14	*Molossusbondae*	20120608_68	Panama, Colon, Gamboa, Ridge	BPBAT059-13	MG191901
15	*Molossusbondae*	20120811_151	Panama, Torti, Iglesia Catolica	BPBAT063-13	MG191810
16	*Molossuscurrentium*	TK 61016	Paraguay, Alto Paraguay	GBGC15983-19	MH185139
17	*Molossuscurrentium*	TK61025	Paraguay	GBGC15984-19	MH185138
18	*Molossusmilleri*	ROM 125964	Cayman Islands		MH938594
19	*Molossusmilleri*	ROM 125963	Cayman Islands		MH449656
20	Molossusm.verrilli*	ROM:MAMM 125388	Dominican Republic	GBMA15534-17	KX355066
21	Molossusm.verrilli*	ROM:MAMM 125386	Dominican Republic	GBMA15535-17	KX355064
22	*Molossussinaloae*	TTU 104174	Mexico, Sinaloa	GBMA22123-19	KX959997
23	*Molossuscoibensis*	ROM:Mamm 122091	Peru	GBMA22156-19	KX960019
24	*Molossuscoibensis*	ROM:Mamm 122130	Peru	GBMA22157-19	KX960020
25	*Molossusrufus*	CMNH68445	Suriname		MH185177
26	*Molossusrufus*	ROM 117466	Suriname, Bakhuis Mountains, Sipaliwini		EU096796
27	*Molossusmolossus*	ROM 104018	Ecuador, Napo, Parque Nacional Yasuni	ABECA036-06	JF448961
28	*Molossusmolossus*	ROM 118785	Ecuador, Orellana	ABECB123-08	JF448972
29	*Promopscentralis*		Outgroup		JF449069
30	*Cynomopsabrasus*		Outgroup		JN312044
31	*Molossopstemminckii*		Outgroup		JF448945
32	*Eumopsauripendulus*		Outgroup		JF454657
33	*Nyctinomopslaticaudatus*		Outgroup		JF447304

**Table 2.** External and cranial measurements (in millimetres) of *Molossusmelini* Specimens from Brazil, Curitiba, Paraná, Atlantic Forest and published data by Montani et al. (2021) and Pavé et al. (2023). Symbols: (-) denotes missing data and (*) indicates specimens with damaged skulls. Values outside parentheses represent the means of the measurements.

Variable	This study		Pavé et al. (2023)	Montani et al. (2021)
Locality	Curitiba city, Paraná state, Brazil		Entre Rios province, Argentina	Melincué Lake, General López Department, Santa Fe Province, Argentina
Sex	3 Females	1 Male	1 Male	2 Males and 1 female
Voucher number	DZUP/CCMZ 2336; 2337; 2338;	DZUP/CCMZ 2339	INALI A635; A651-653	MG-ZV-M 338
Total length (ToL)	93.5 (3; 91.65-94.82)	102.71	105	(2; 116-119)
Tail length (TL)	31.1 (3; 28.98-32.94)	35.36	34	(2; 42.5-48)
			♂ 35.5 (2; 34-36.9)	
			♀ 37.2 (2; 36-38.4)	
Hindfoot length (HFL)	8.5 (3; 7.74-8.86)	7.96	7	(2; 7-8)
Ear length (EL)	11.6 (2*; 11.42-11.92)	10.15	13	(2; 13.5-14.5)
Forearm length (FA)	39.9 (3; 39.45-40.27)	40.08	41.5	(3; 41.5-43.6)
			♂ 41.9 (2; 41.5-42.3)	
			♀ 41.6 (4; 41.3-42.5)	
Greatest length of skull including incisors (GLS)	17.6 (2*; 17.62-17.64)	18.07	19.5	(2; 18.4-19.3)
Condylobasal length (CBL)	16.8 (2*; 16.78-16.97)	16.45	17.1	(2; 17.3-17.9)
Postorbital constriction (PC)	4.2 (2*; 4.18-4.21)	4.03	4.2	(2; 4.0-4.2)
Zygomatic breadth (ZB)	-*	-*	12.3	(2; 12.4-12.6)
Breadth of braincase (BB)	8.9 (2*; 8.94-8.99)	9.59	9.5	(2; 9.7-9.8)
Mastoideal breadth (MB)	10.4 (2*; 10.17-10.74)	11.10	11.8	(2; 12.0-12.1)
Length of maxillary toothrow (LMxT)	6.1 (2*; 6.02-6.31)	6.27	6.7	(2; 6.7-6.8)
Palatal length (PL)	5.66 (2*; 5.51-5.82)	5.68	6.2	(2; 6.6-6.9)
Width across canines (C–C)	4.48 (2*; 4.25-4.71)	4.56	4.9	(2; 5.0-5.0)
Width across molars (M2–M2)	7.5 (2*; 7.14-7.89)	7.83	8.3	(2; 8.3-8.3)
Height of the sagittal crest (SAR)	1 (2*; 0.82-1.20)	1.03	1.3	(2; 1.1-1.5)
Length of mandible (LM)	12.1 (2*; 11.98-12.33)	12.62	13.5	(2; 13.2-13.3)
Length of mandibular toothrow (LMdT)	6.87 (2*; 6.74-7.00)	6.97	7.9	(2; 7.4-7.6)
