# Supplementary figures and images for: Molossusmelini Montani et al. 2021 (Chiroptera, Molossidae) in Brazil: new insights for distribuition, morphology and genetics
Source: Biodivers Data J. 2024 Feb 12;12:e114261. doi: 10.3897/BDJ.12.e114261 (PMC10880027; doi:10.3897/BDJ.12.e114261)

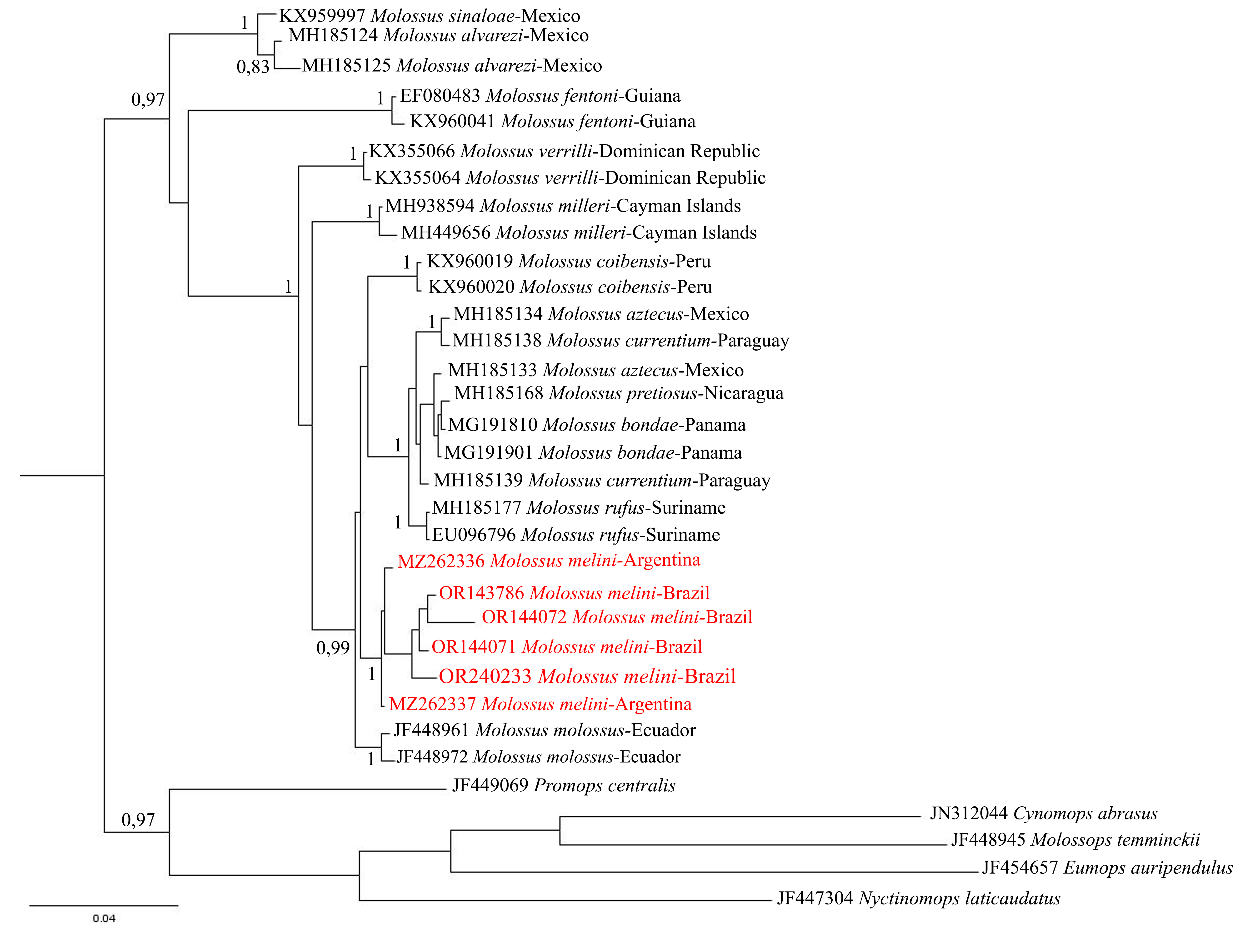

Supplement: Supplementary material 1 — Bayesian Inference [file bdj-12-e114261-s001.jpg]
